# Variations in the financial impact of the COVID-19 pandemic across 5 continents: A cross-sectional, individual level analysis

**DOI:** 10.1016/j.eclinm.2022.101284

**Published:** 2022-01-28

**Authors:** Aditya K Khetan, Salim Yusuf, Patricio Lopez-Jaramillo, Andrzej Szuba, Andres Orlandini, Nafiza Mat-Nasir, Aytekin Oguz, Rajeev Gupta, Álvaro Avezum, Ismail Rosnah, Paul Poirier, Koon K Teo, Andreas Wielgosz, Scott A. Lear, Lia M. Palileo-Villanueva, Pamela Serón, Jephat Chifamba, Sumathy Rangarajan, Maha Mushtaha, Deepa Mohan, Karen Yeates, Martin McKee, Prem K Mony, Marjan Walli-Attaei, Hamda Khansaheb, Annika Rosengren, Khalid F Alhabib, Iolanthé M Kruger, María-José Paucar, Erkin Mirrakhimov, Batyrbek Assembekov, Darryl P Leong

**Affiliations:** a Population Health Research Institute, McMaster University and Hamilton Health Sciences, Hamilton, ON L8L 2X2, Canada; b Medical School, Universidad de Santander (UDES), Masira Research Institute, Bucaramanga, Colombia; c Department of Angiology, Hypertension and Diabetology, Wroclaw Medical University, Wroclaw, Poland; d ECLA (Estudios Clínicos Latino America) Instituto Cardiovascular de Rosario, Argentina; e Faculty of Medicine, Universiti Teknologi MARA, Selangor, Malaysia; f Faculty of Medicine, Istanbul Medeniyet University, Istanbul, Turkey; g Eternal Heart Care Center and Research Institute, Jaipur, India; h International Research Center, Hospital Alemão Oswaldo Cruz, São Paulo, Brazil; i Faculty of Medicine of UKM, UKM Medical Center, Kuala Lumpur, Malaysia; j Faculté de pharmacie, Université Laval, Institut universitaire de cardiologie et de pneumologie de Québec, Québec, Canada; k University of Ottawa Heart Institute, Ottawa, ON, Canada; l Faculty of Health Sciences, Simon Fraser University, Vancouver, British Columbia, Canada; m UP College of Medicine, University of the Philippines Manila, Manila, Philippines; n Faculty of Medicine, Universidad de La Frontera, Claro Solar, Temuco, Chile; o Department of Biomedical Sciences, Physiology Unit, Faculty of Medicine and Sciences, University of Zimbabwe, Zimbabwe; p Department of Epidemiology, Madras Diabetes Research Foundation, Chennai, India; q Department of Medicine, Queen's University, Kingston, ON, Canada; r London School of Hygiene and Tropical Medicine, London, United Kingdom; s St John's Medical College & Research Institute, Bangalore, India; t Dubai Health Authority, Dubai, United Arab Emirates; u Sahlgrenska Academy, University of Gothenburg and Sahlgrenska University Hospital, VGR region, Sweden; v King Fahad Cardiac Center, College of Medicine, King Saud University, Riyadh, Saudi Arabia; w Potchefstroom Campus, Africa Unit for Transdisciplinary Health Research (AUTHeR), North-West University, South Africa; x Facultad de Ciencias de la Salud Eugenio Espejo, Universidad UTE, Quito, Ecuador; y Kyrgyz State Medical Academy, Bishkek, Kyrgyzstan; z Al-Farabi Kazakh National University, Almaty, Kazakhstan

## Abstract

**Background:**

COVID-19 has caused profound socio-economic changes worldwide. However, internationally comparative data regarding the financial impact on individuals is sparse. Therefore, we conducted a survey of the financial impact of the pandemic on individuals, using an international cohort that has been well-characterized prior to the pandemic.

**Methods:**

Between August 2020 and September 2021, we surveyed 24,506 community-dwelling participants from the Prospective Urban-Rural Epidemiology (PURE) study across high (HIC), upper middle (UMIC)-and lower middle (LMIC)-income countries. We collected information regarding the impact of the pandemic on their self-reported personal finances and sources of income.

**Findings:**

Overall, 32.4% of participants had suffered an adverse financial impact, defined as job loss, inability to meet financial obligations or essential needs, or using savings to meet financial obligations. 8.4% of participants had lost a job (temporarily or permanently); 14.6% of participants were unable to meet financial obligations or essential needs at the time of the survey and 16.3% were using their savings to meet financial obligations. Participants with a post-secondary education were least likely to be adversely impacted (19.6%), compared with 33.4% of those with secondary education and 33.5% of those with pre-secondary education. Similarly, those in the highest wealth tertile were least likely to be financially impacted (26.7%), compared with 32.5% in the middle tertile and 30.4% in the bottom tertile participants. Compared with HICs, financial impact was greater in UMIC [odds ratio of 2.09 (1.88–2.33)] and greatest in LMIC [odds ratio of 16.88 (14.69–19.39)]. HIC participants with the lowest educational attainment suffered less financial impact (15.1% of participants affected) than those with the highest education in UMIC (22.0% of participants affected). Similarly, participants with the lowest education in UMIC experienced less financial impact (28.3%) than those with the highest education in LMIC (45.9%). A similar gradient was seen across country income categories when compared by pre-pandemic wealth status.

**Interpretation:**

The financial impact of the pandemic differs more between HIC, UMIC, and LMIC than between socio-economic categories within a country income level. The most disadvantaged socio-economic subgroups in HIC had a lower financial impact from the pandemic than the most advantaged subgroup in UMIC, with a similar disparity seen between UMIC and LMIC. Continued high levels of infection will exacerbate financial inequity between countries and hinder progress towards the sustainable development goals, emphasising the importance of effective measures to control COVID-19 and, especially, ensuring high vaccine coverage in all countries.

**Funding:**

Funding for this study was provided by the Canadian Institutes of Health Research and the International Development Research Centre.

**Research in context**

**Evidence before this study**

We searched PubMed for relevant research published between March 1, 2020, and October 1, 2021, using the term “financial impact” AND “COVID-19″ AND “individual level”. We screened papers by title and abstract to identify full-text reports that were relevant to the study aims. Previous studies have looked at individual level financial impact of the COVID-19 pandemic within a single country, or a small group of countries. However, these estimates of the financial effects of the pandemic cannot be directly compared, given differences in data collection and sampling methods. Moreover, these surveys usually did not characterize participants prior to the onset of the pandemic.

**Added value of this study**

We conducted a global survey of the financial impact of the pandemic on individuals, using a cohort that has been well-characterized prior to the pandemic, with information collected in a standardized manner across countries. Globally, nearly one third of participants suffered an adverse financial impact of the COVID-19 pandemic. We show that the primary inequity in the financial impact of the pandemic is between rich and poor countries, and that this difference is less marked within individuals at the same country-income level.

**Implications of all the available evidence**

With COVID-19, large economic gaps have arisen between individuals living in lower income countries and those living in higher income countries. This wide financial inequity between countries has implications for future global health, international peace, and migration. Expanding vaccine coverage in low- and middle-income countries will likely have the largest effect in decreasing the current health and economic gap between nations.

Alt-text: Unlabelled box

## Introduction

Since March 2020, COVID-19 is reported as having caused >4 million deaths globally, though the real numbers may be substantially higher, with the unrecorded toll likely to be especially great in lower income countries.^1^ However, both the disease and the responses necessary to reduce its spread have caused profound social and economic damage on a scale almost unprecedented in peacetime. Understanding the scale and nature of this damage is essential to minimizing the effects of the pandemic.

Globally, Gross Domestic Product (GDP) is estimated to have contracted by 4.3% in 2020.^2^ However, GDP does not capture the economic impact on individuals; it fails to capture the distribution of income or the support that people can draw on.^3^ Subjective measures of financial conditions offer an alternative that correlate well with measures of health and wellbeing, and are especially helpful in cross-country comparisons.^4^^,^^5^ We therefore aimed to survey the financial impact of the pandemic on individuals in the community irrespective of whether they contracted COVID in a standardized manner, within an ongoing global cohort that has been well-characterized prior to the pandemic.

Previous research points to wide variations in financial impacts of the pandemic within countries by race, ethnicity and a range of socio-economic characteristics.^6^ However, how these factors operate in countries with different income levels is unknown given the lack of standardized individual-level data across countries. We hypothesized that (1) the financial impact would be worse in poorer countries and within given country income level, the financial impact of the pandemic will be greater amongst disadvantaged socioeconomic subgroups (defined by wealth or education), and (2) individuals with the highest wealth and education, and thus access to resources that confer resilience, will be relatively financially unaffected by the pandemic.^7^ Given that high-income countries rapidly rolled out large relief and stimulus packages, while lower income countries, with large informal sectors and limited resources, had considerably more modest relief packages, we hypothesized that individuals living in lower income countries would suffer a larger financial impact.^8^ Understanding differences in financial impact of the pandemic by country-income level can help facilitate global governance measures, including measures aimed at controlling the ongoing pandemic, that narrow socioeconomic gaps between countries.

## Methods

### Study design and participants

The Prospective Urban-Rural Epidemiology (PURE) study is a prospective cohort study of community-based adults recruited at an age of 35–70 years across six geographical regions: Asia, Africa, Europe, South America, North America, and the Middle East. The design of the PURE study has been described previously.^9^ At the start of the study in 2003, countries were selected to reflect diverse socioeconomic conditions. Due to feasibility considerations, we did not undertake proportionate sampling of all countries worldwide, or of regions within countries. However, we enrolled individuals from both urban and rural communities and once communities for inclusion were identified, we utilized an unbiased sampling approach. Prior analyses have shown only minor differences between the PURE population and national data for key indicators.^9^^,^^10^

At the start of the study, households were eligible if at least one member was aged 35–70 years and if household members intended to stay at that address for another four years. The PURE study was approved by the relevant research ethics committees in all participating countries and sites. All participants provided written informed consent. The study was coordinated by the Population Health Research Institute, Hamilton Health Sciences and McMaster University, Hamilton, ON, Canada. The manuscript is adherent to STROBE guidelines.

### Procedures

Standardized methods were used to collect baseline information. A questionnaire, administered by trained study personnel, elicited self-reported demographics, education levels and occupation. Wealth, calculated at the household level, was defined by an index on the basis of ownership of assets and housing characteristics.^11^ This index has been previously validated in low-, middle- and high-income countries, and documented to be a robust measure of wealth, consistent with measures of income and expenditure.^12^^,^^13^ Participants were categorized into country-specific tertiles based on their wealth percentile, with higher categories denoting greater wealth relative to others from the same country in lower wealth categories.

The first case of COVID-19 was reported in China in December 2019 and the COVID-19 pandemic was declared by the World Health Organization on March 11, 2020. Between August 2020 and September 2021, in an ongoing effort to better understand the epidemiology and impact of COVID-19, we invited 25,927 community-dwelling PURE participants from 16 countries to participate in a survey, of whom 1421 (5.8%) declined. Information was collected through in-person visits where possible, and by telephone otherwise. Most visits were completed over the telephone (76.9%), with the remainder conducted in-person. In participating sites and countries, all PURE participants were eligible for this substudy, with data collected at the individual participant level. We collected information on whether they had been diagnosed with COVID-19, and the cumulative impact of the pandemic on their self-reported personal finances and sources of income. Participants were asked, ‘How has the COVID pandemic impacted your personal finances or source of income?’ They were asked if they had lost their job or main source of income, either on a temporary or permanent basis (categorized as ‘lost job’). In addition, they were asked if they were unable to meet financial obligations or essential needs. This included rent, mortgage payments, groceries or electricity bills (categorized as ‘unable to meet financial obligations’). Thirdly, they were asked if they were using their savings to meet financial obligations (categorized as ‘using savings’). Participants who suffered an adverse financial effect that was not included in the categories above could select the option of ‘other’. This primarily included individuals who had reduced income and/or work. Participants were advised to select all options that were applicable to them. Participants who experienced any of ‘lost job’, ‘unable to meet financial obligations’, ‘using savings’ or other financial adverse effects (such as reduced income or work) were considered to be ‘financially impacted’.

Given wide disparities in vaccination rates between countries, and the expectation that high vaccine coverage is crucial to the economic recovery of nations, we analyzed the relationship of country level vaccination rates with the financial impact of the pandemic.^14^ We used the Our World in Data COVID-19 vaccination dataset, a global public dataset that tracks the scale and rate of the vaccine rollout across the world.^15^ Vaccination rates are the proportion of a country's total population that received at least 1 dose of a COVID-19 vaccine (as of 11th October 2021).

Given that GDP has numerous shortcomings, we used the 2020 Social Progress Index (SPI) as a measure of a country's human progress.^16^ The SPI measures three dimensions of society: 1) basic human needs (e.g., food, water, and shelter), 2) foundations of wellbeing (e.g., education and health), and 3) the chance to pursue opportunities (e.g., access to knowledge, freedom from discrimination).

### Statistical analysis

For this analysis, we categorized countries according to the World Bank country-income classification in 2020. Countries were categorized as high-income (HIC- Canada, Sweden, United Arab Emirates, Chile, Poland, and Saudi Arabia), upper middle-income (UMIC- Argentina, Malaysia, Brazil, Colombia, South Africa, and Turkey) and lower middle-income (LMIC- Philippines, India, Tanzania, and Zimbabwe). We did not have any low-income countries in our sample.

Bar charts were used to visualize proportions. Proportions were compared using Chi-squared tests. Scatterplots were used to visualize the relationship between the proportion of a country's participants who were financially impacted and a) SPI; b) Gross National Income (GNI) per capita; c) the proportion of the country's population that was vaccinated. Pearson correlation coefficients were used to measure the strengths of correlation. To identify characteristics that were independently associated with the odds of being financially impacted, we used a multilevel mixed-effects logistic regression model. We used a two-level model, in which individuals were nested in communities. Exposures were country income level, individual-level wealth, individual-level education, and occupation. We adjusted for age, sex, and baseline disease burden. Baseline disease burden was defined as the total number of chronic illnesses that an individual had, and included myocardial infarction, stroke, heart failure, Chronic Obstructive Pulmonary Disease (COPD), and cancer.

We used STATA 16.1 software for the analyses.

### Role of the funding source

The funder of the study had no role in study design, data collection, data analysis, data interpretation, or writing of the report. All authors had full access to the data in the study and had final responsibility for the decision to submit for publication.

## Results

### Baseline characteristics

Baseline characteristics of participants were collected at their entry into the PURE study and are shown in Table 1. There were 24,506 participants, with 7712 from HIC (age 64.3 ± 10.1 years, 58.2% female), 9038 from UMIC (63.2 ± 9.3 years, 61.9% female) and 7756 from LMIC (62.0 ± 9.2 years, 59.4% female). More participants in HIC had completed post-secondary education and were in a professional/managerial occupation.

**Table 1:** **Baseline Characteristics.** Categorical variables are presented as counts (column percentage). Age as of December 2020 is presented as mean ± standard deviation (SD). Household size shows the median (25th – 75th percentile) number of household inhabitants.

	Overall (*n* = 24,506)	HIC (*n* = 7712)	UMIC (*n* = 9038)	LMIC (*n* = 7756)
Mean age (years)	63.2 ± 9.6	64.3 ± 10.1	63.2 ± 9.3	62.0 ± 9.2
Female	14,688 (59.9)	4490 (58.2)	5593 (61.9)	4605 (59.4)
**Education Level**				
Pre-secondary school	10,669 (43.7)	1256 (16.3)	4690 (51.9)	4723 (61.4)
Secondary school	7392 (30.3)	2527 (32.8)	2584 (28.6)	2281 (29.6)
Post-secondary school	6371 (26.1)	3921 (50.9)	1757 (19.5)	693 (9.0)
**Occupation**				
Professionals/managers	4781 (23.9)	3084 (40.1)	1409 (15.7)	288 (8.7)
Skilled workers	5908 (29.6)	2361 (30.7)	2654 (29.5)	893 (27.0)
Unskilled workers	3528 (17.7)	1165 (15.2)	1377 (15.3)	986 (29.8)
Homemakers	5764 (28.9)	1077 (14.0)	3550 (39.5)	1137 (34.4)
Household Size	5 (4–7)	4 (5–7)	6 (4–8)	5 (4–6)
Diagnosed with COVID-19	1069 (4.4)	444 (5.8)	560 (6.2)	65 (0.8)
Baseline Disease Burden	0.08±.29	0.15±.40	0.08±.30	0.04±.21

### Financial impact

Overall, 32.4% of participants had suffered an adverse financial impact. 8.4% of participants in our study lost a job, either on a temporary or permanent basis; 14.6% of participants were unable to meet financial obligations or essential needs at the time of the survey and 16.3% were using their savings to meet financial obligations.

### Differences in financial impact by country income level

**Figure 1:**
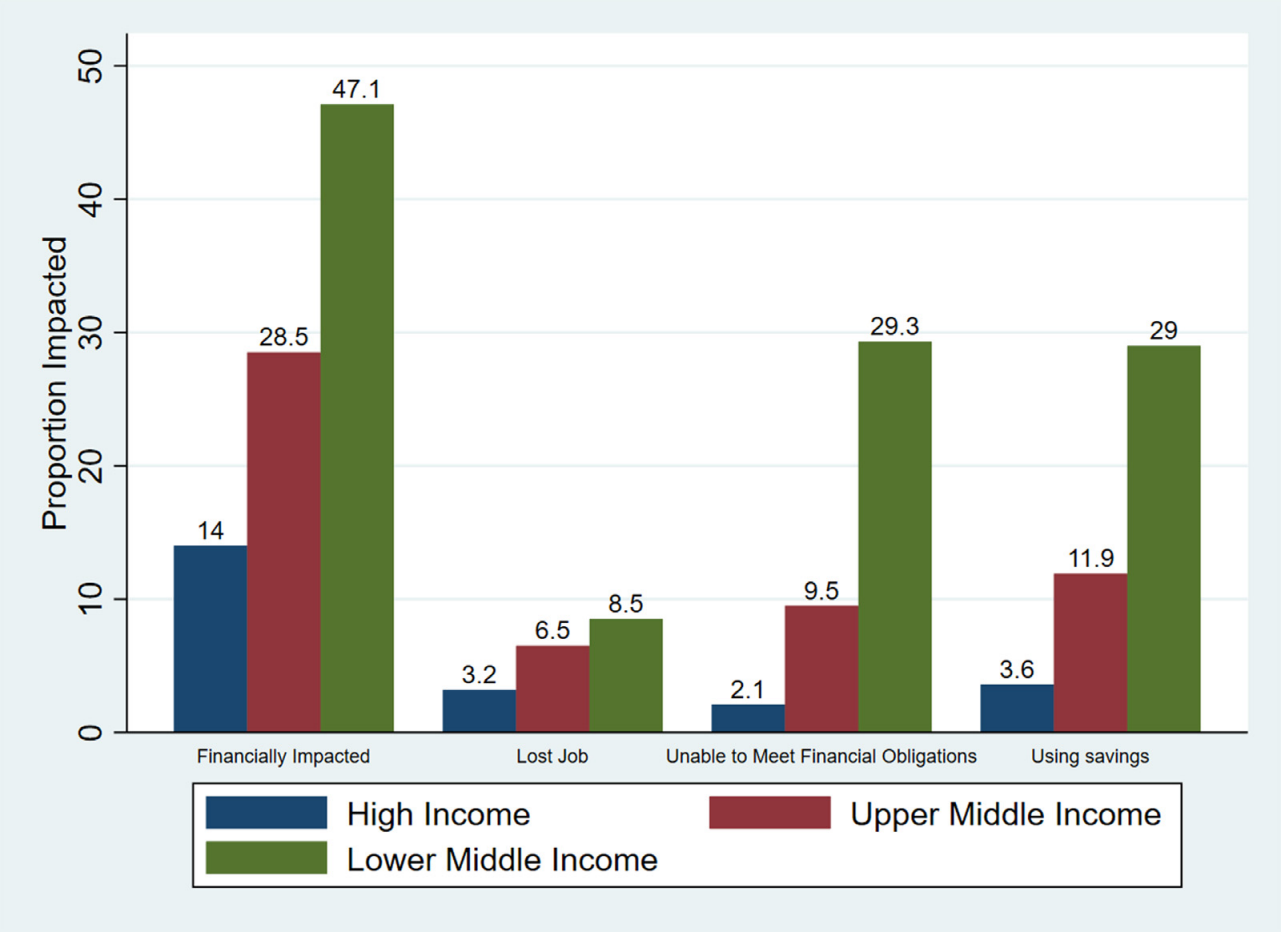
Financial Impact of COVID-19, by Country Income Category Financially impacted includes those who lost a job (either on a temporary or permanent basis), were unable to meet financial obligations or essential needs, were using savings to meet financial obligations or suffered other financial adverse effects. Other financial adverse effects primarily included reduced work hours and/or income. Individuals selected all financial adverse effects that were applicable to them.

**Figure 2:**
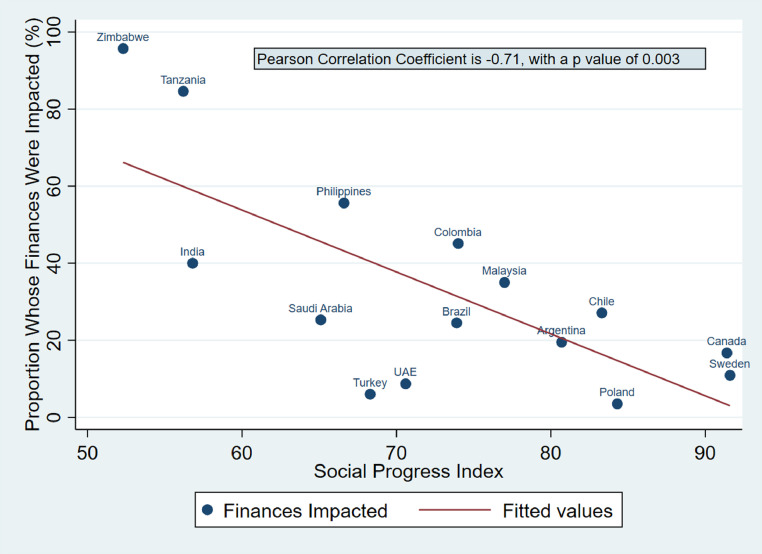
Scatterplot of Social Progress Index versus Proportion Whose Finances Were Adversely Impacted, by Country South Africa excluded from this figure as it had <100 participants.

Financial impact, by country income level, is shown in Figure 1. There were large differences between country income levels, with the proportion of participants financially impacted in LMIC more than three times the proportion financially impacted in HIC (47.1% vs 14%, absolute difference 33.1%, 95% CI 31.7% to 34.5%, *p* value <0.0001). Figure 2 shows a scatterplot of Social Progress Index and proportion of participants in a country who were financially impacted. There was a strong inverse correlation between the two, with a Pearson correlation coefficient of −0.71 (95% CI −0.31 to −0.90, *p* = 0.003), indicating the lower the country's social development, the greater the financial impact of the pandemic. The country with the largest financial impact was Zimbabwe, with 95.7% of participants financially impacted. Conversely, Poland had the least financial impact, as only 3.5% of participants suffered an adverse financial impact of the COVID-19 pandemic. The Pearson correlation coefficient for the relationship between GNI per capita and proportion in a country whose finances were adversely impacted was −0.58 (95% CI −0.09 to −0.84, *p* = 0.02). A scatterplot of this association demonstrates a similar pattern to the relationship between the SPI and the proportion financially impacted (Supplementary Figure 1). Supplementary Figure 2 shows a scatterplot of GNI per capita versus vaccination rates in each country. The Pearson correlation coefficient is 0.61, with a *p* value of 0.02. Supplementary Figure 3 shows a scatterplot of vaccination rates in each country versus the proportion of the country's participants who were financially impacted. There is a strong inverse correlation between the two variables, with a Pearson correlation coefficient of −0.82 (95% CI −0.51 to −0.95, *p* = 0.0004). This indicates that a high national COVID-19 vaccination rate was strongly associated with lower individual-level financial impact.

### Differences in financial impact within a country income level

**Figure 3:**
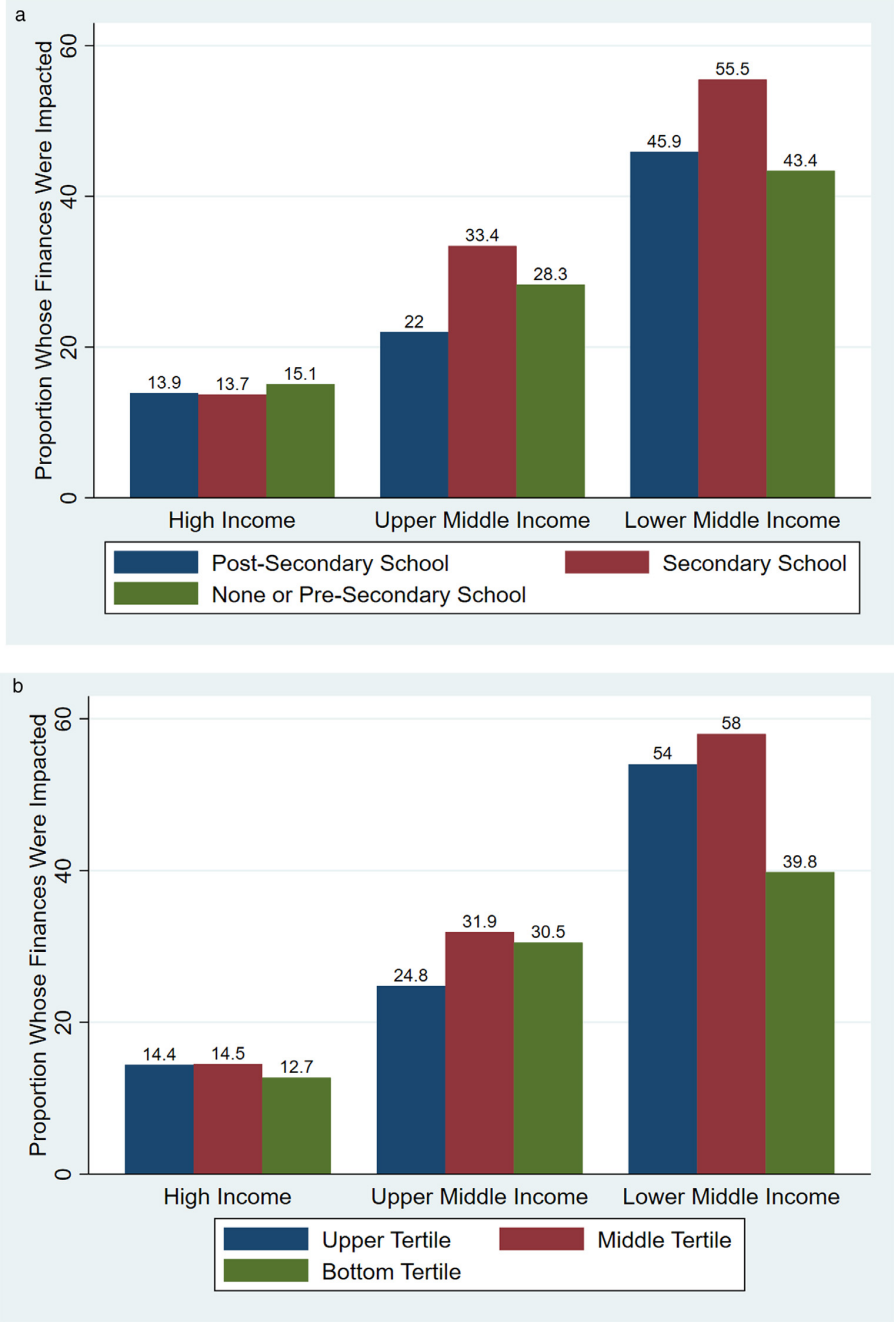
A- Proportion Impacted Financially by COVID-19, by Education For difference between ‘None or Pre-Secondary School’ in ‘High Income’ and ‘Post Secondary School’ in ‘Upper Middle Income’, *p* value is <0.0001. Similarly, for difference between ‘None or Pre-Secondary School’ in ‘Upper Middle Income’ and ‘Post Secondary School’ in ‘Lower Middle Income’, *p* value is <0.0001. B- Proportion Impacted Financially by COVID-19, by Pre-pandemic Wealth For difference between ‘Bottom Tertile’ in ‘High Income’ and ‘Upper Tertile’ in ‘Upper Middle Income’, *p* value is <0.0001. Similarly, for difference between ‘Bottom Tertile’ in ‘Upper Middle Income’ and ‘Upper Tertile’ in ‘Lower Middle Income’, *p* value is <0.0001.

Within each country income level, differences in the financial impact of COVID-19 were assessed by education and pre-pandemic wealth. In the global cohort, those with a post-secondary education had the smallest proportion financially impacted (19.6%), with 33.4% of those with a secondary education financially impacted and 33.5% of those with a pre-secondary education financially impacted. Figure 3A shows the proportion impacted financially by the pandemic, by education and country-income level. Fewer participants in HIC with the lowest educational attainment (15.1%) suffered financial impact than participants with the highest educational attainment in UMIC (22.0%), *p*<0.0001. Similarly, fewer participants in UMIC with the lowest educational attainment suffered financial impact (28.3%) than participants with the highest educational attainment in LMIC (45.9%), *p*<0.0001. In the global cohort, those in the highest wealth tertile had the smallest proportion financially impacted (26.7%), with 32.5% of middle tertile participants financially impacted and 30.4% of bottom tertile participants financially impacted. Figure 3B shows the proportion impacted financially by the pandemic, by wealth (measured prior to the pandemic) and country-income level. A similar pattern as education is seen, with the poorest participants in HIC less impacted financially than the wealthiest participants in UMIC (*p*<0.0001); and the poorest participants in UMIC less financially impacted than the wealthiest in LMIC (*p*<0.0001).

Factors independently associated with adverse financial effects from the pandemic

We modelled the odds of being financially impacted by the pandemic, using a multilevel logistic regression model (Table 2). Compared with those with a post-secondary school education, those with a secondary school education had higher odds of being financially impacted (OR 1.27, 95% CI 1.14–1.42). For homemakers, compared with professionals/managers, the odds ratio for being financially impacted was 1.21 (1.06–1.39). Compared with the wealthiest third, those in the poorest third had an odds ratio of being financially impacted of 1.14 (1.03–1.26). For country-income category, however, the difference in odds was starker. Compared with HIC participants, UMIC participants had an odds ratio of 2.09 (1.88–2.33) for being financially impacted while LMIC participants had an odds ratio of 16.88 (14.69–19.39) for being financially impacted. The Intraclass Correlation Coefficient (ICC) at the community level was 0.28 (95% CI 0.24–0.34).

**Table 2:** Multi-level logistic regression of the odds of being financially impacted.

Pre-pandemic Characteristic	OR (95% CI)	*p*- value
***Country Income Level***		
**HIC**	1	
**UMIC**	2.09 (1.88–2.33)	<0.001
**LMIC**	16.88 (14.69–19.39)	<0.001
***Education***		
**Post-Secondary**	1	
**Secondary**	1.27 (1.14–1.42)	<0.001
**Pre-Secondary**	1.09 (0.96–1.23)	0.2
***Wealth***		
**Wealthiest Tertile**	1	
**Middle Tertile**	1.12 (1.03–1.23)	0.01
**Bottom Tertile**	1.14 (1.03–1.26)	0.01
***Occupation***		
**Professionals/Managers**	1	
**Skilled workers**	1.11 (0.98–1.25)	0.09
**Unskilled workers**	1.21 (1.05–1.40)	0.008
**Homemakers**	1.21 (1.06–1.39)	0.006

OR- Odds Ratio. Model is adjusted for age, sex, baseline disease burden and variables listed above.

In a multilevel model that adjusted for age, sex, education, wealth, occupation, country income category and baseline disease burden, compared to participants who did not have a diagnosed COVID-19 infection, participants diagnosed with COVID-19 had a slightly higher odds of being financially impacted (1.08, 1.00–1.17, *p* value 0.06).

## Discussion

Our study has several important findings. First, we show the primary difference in the financial impact of the pandemic is between countries of different income levels, and that this difference is less marked within individuals at the same country-income level. Strikingly, the most disadvantaged socio-economic subgroup in HIC had a smaller proportion of participants economically impacted than the most advantaged economic subgroup in UMIC. Along the same lines, the most advantaged economic subgroup in LMIC had a greater proportion of participants financially impacted than the least advantaged economic subgroup in UMIC, thus emphasizing that the primary inequality is not within countries, but between countries. In recent years, there has been increasing socio-political focus in HIC on ‘levelling up’ disadvantaged regions and communities.^17^ Over time, this may result in a decrease in within-country inequality in high income countries.^18^ On the other hand, as seen in our study, the adverse economic impact of COVID-19 in lower income countries also extends to traditionally advantaged communities (those with higher education attainment and/or more wealth). Therefore, we may see a paradoxical decrease in inequality in lower income countries too, as these countries see millions of people leave their recent middle-class prosperity behind, resulting in historically rich communities becoming poorer in these countries.^19^ As a result, inequity between countries will likely rise, with implications for global governance, global health, international peace and migration. Finally, the reasons why those in the lowest education and wealth tertile (particularly in LMIC) fared less poorly than those in the middle tertile are not immediately clear. One possibility is that the former were more likely to be employed in essential sectors, but this is speculative.

Second, vaccine inequity closely correlates with inequity in financial impact, with the same countries that are more impacted financially also having the smallest proportion of their population vaccinated. Given that high vaccine coverage is a vital route to an economy's recovery post COVID-19, the gap in vaccination will likely increase the economic gap between higher income and lower income countries- exaggerating the large gap that already exists. Models suggest that all countries would benefit economically if middle and lower-income countries had equal access to a COVID-19 vaccine, with high-income countries expected to get back $4.80 for every $1 spent on supplying vaccines.^20^ Decreasing vaccine inequity should be a top priority for high-income countries and global governance organizations, in order to mitigate the socio-economic impact of COVID-19 in middle and low-income countries. This will require a package of measures, addressing production, allocation, availability, deployment and vaccine hesitancy, but especially a level of political commitment that has so far been lacking.^21^

Estimates of adverse financial impact in our study match well with estimates from other sources. For instance, longitudinal household survey data from Ethiopia, Malawi, Nigeria and Uganda showed that an estimated 77% of the population in these countries lost income during the pandemic.^22^ Similarly, in India, nationally representative data showed that the lockdown adversely impacted 43% of the national workforce.^23^ In Canada, periodic labor force surveys from July 2020 to February 2021 showed that between 20 and 22% of people lived in households that reported it was “difficult” or “very difficult” to meet basic household financial commitments in the last four weeks.^24^ These reported estimates of the financial effects of the pandemic cannot, however, be directly compared, given differences in data collection and sampling methods. Our study adds important new information because of the standardized way in which participants were sampled and the prospective collection of data.

The Social Progress Index correlated more strongly than GNI per capita with the financial impact of COVID-19 within a country. This suggests that at a given national income, countries with greater social development were more resilient to the adverse financial impacts of the pandemic. This is unsurprising; governments choose how to spend their money and it is intuitive that those that prioritise social development will provide greater protection for their population. This is consistent with evidence from the global financial crisis at the end of the 2000s. This finding underscores the importance of social safety nets as part of preparedness for major health threats.^25^

The socio-economic impact of COVID-19 goes beyond financial impact, and includes education and food insecurity. Both these factors could further widen the socio-economic gap between countries. According to UNICEF estimates, at least 463 million students were unable to access remote learning modalities. By March 2021, 168 million children globally had been unable to go to school for almost an entire year. A disproportionate share of these children live in middle and low-income countries.^26^ Given that higher educational attainment is strongly associated with future income and productivity, a disproportionate decline in the educational attainment of children in middle and low-income countries can widen the future economic gap between nations.^27^

The strength of our study is that it has a global population that has been well-characterized prior to the pandemic, with standardized data collection methods across countries. This enables robust cross-country comparisons with individual level data, while reducing the possibility of bias. There are also some limitations. One is a lack of low-income countries in our sample. However, the findings of the lowest income countries in our study (Tanzania, Zimbabwe) can likely be extended to several low-income countries, particularly since >95% of the study population in Zimbabwe had suffered a financial adverse effect since the start of the pandemic. Another is our inability to characterise the scale of the pandemic as it affected each country, and how the pandemic's effect varied over the duration of the data collection for this study. While data on incidence and deaths are available, there are major concerns about their accuracy in many countries, whether due to weaknesses in disease surveillance and vital registration systems or other reasons.^28^,^29^ Third, the mean age of our study population in December 2020 was 63 years; the implications of our findings for younger people are uncertain. Fourth, we assessed financial impact in a qualitative manner, which did not capture the severity of the financial impact. For instance, we did not capture duration of job loss, or extent of savings depreciation. Finally, we could not assess the effect or role of extreme individual level wealth inequality, as between March 2020 and March 2021, the world's 2365 billionaires saw their wealth rise by USD 4 trillion, a relative increase of 54%.^30^ Further research should examine the role of government policy in mitigating or exacerbating financial impact, including policies aimed at decreasing mobility, stimulus programs, subsidies and other forms of aid.

In a diverse cohort from 16 countries, we show that the primary difference in the financial impact of the pandemic on individuals is related to differences in the income levels of their countries, rather then their individual levels of wealth or education. We show that the most disadvantaged socio-economic subgroup in HIC was less economically impacted than the most advantaged economic subgroup in UMIC, while the most advantaged socio-economic subgroup in LMIC was more financially impacted than the least advantaged socio-economic subgroup in UMIC. Globally, increasing vaccine coverage in all countries should be a top priority to mitigate the disproportionate socio-economic impact of COVID-19 in middle and low-income countries.

## Declaration of interests

Darryl P Leong is supported by International Development Research Centre Project grant 109,556, Canadian Institutes of Health Research 177,736 and the Heart and Stroke Foundation of Canada. Andrzej Szuba is supported by Polish Ministry of Science and Higher Education (Grant: MNiSW- Nr290/W-PURE/2008/0) and PHRI (Grant). Álvaro Avezum is supported by EMS– Research Funding for COVID-19 Initiated Investigator Study and Bayer– Research Funding for COVID-19 Initiated Investigator Study. Andreas Wielgosz is Member, Board of Directors, InterAmerican Heart Foundation. Other authors did not declare any interests.

## Appendix Supplementary materials

Image, application 1

Image, application 2
