# Supplementary material for: Variations in the financial impact of the COVID-19 pandemic across 5 continents: A cross-sectional, individual level analysis
Source: eClinicalMedicine. 2022 Jan 28;44:101284. doi: 10.1016/j.eclinm.2022.101284 (PMC8794545; doi:10.1016/j.eclinm.2022.101284)
Supplement: Supplementary file 1 [file mmc1.doc]

**Funding/Support:**

Dr S Yusuf is supported by the Marion W Burke endowed chair of the Heart and Stroke Foundation of Ontario.

The PURE study is an investigator-initiated study that is funded by the Population Health Research Institute, Hamilton Health Sciences Research Institute (HHSRI), the Canadian Institutes of Health Research, Heart and Stroke Foundation of Ontario, Support from Canadian Institutes of Health Research’s Strategy for Patient Oriented Research, through the Ontario SPOR Support Unit, as well as the Ontario Ministry of Health and Long-Term Care and through unrestricted grants from several pharmaceutical companies [with major contributions from AstraZeneca (Canada), Sanofi-Aventis (France and Canada), Boehringer Ingelheim (Germany and Canada), Servier, and GlaxoSmithKline], and additional contributions from Novartis and King Pharma and from various national or local organizations in participating countries.

**PURE SARS-CoV-2: A Prospective Urban Rural Epidemiology (PURE) Sub-study, work was carried out with the aid of a grant from the International Development Research Centre, Ottawa, Canada (CIHR_COVID_GA_06-2020E)**

These include: **Argentina:** Fundacion ECLA **(Estudios Clínicos Latino America)** ; **Bangladesh**: Independent University, Bangladesh and Mitra and Associates; **Brazil:** Hospital Alemão Oswaldo Cruz, São Paulo, Brazil; **Canada:** This study was supported by an unrestricted grant from Dairy Farmers of Canada and the National Dairy Council (U.S.), Public Health Agency of Canada and Champlain Cardiovascular Disease Prevention Network; **Chile:** Universidad de La Frontera [DI13-PE11]; **China:** National Center for Cardiovascular Diseases and ThinkTank Research Center for Health Development; **Colombia:** Colciencias (grant 6566-04-18062 and grant 6517-777-58228); **India:** Indian Council of Medical Research; **Malaysia:** Ministry of Science, Technology and Innovation of Malaysia (grant number: 100-IRDC/BIOTEK 16/6/21 [13/2007], and 07-05-IFN-BPH 010), Ministry of Higher Education of Malaysia (grant number: 600-RMI/LRGS/5/3 [2/2011]), Universiti Teknologi MARA, Universiti Kebangsaan Malaysia (UKM-Hejim-Komuniti-15-2010); **occupied Palestinian territory:** the United Nations Relief and Works Agency for Palestine Refugees in the Near East, occupied Palestinian territory; International Development Research Centre, Canada; **Philippines:** Philippine Council for Health Research and Development; **Poland:** Polish Ministry of Science and Higher Education (grant number: 290/W-PURE/2008/0), Wroclaw Medical University; **Saudi Arabia:** Saudi Heart Association, Dr.Mohammad Alfagih Hospital, The Deanship of Scientific Research at King Saud University (Research group number: RG -1436-013), Riyadh; Saleh Hamza Serafi Chair for Research of Coronary Heart Disease, Umm AlQura University, Makkah, Saudi Arabia; **South Africa:** The North-West University, SA and Netherlands Programme for Alternative Development, National Research Foundation, Medical Research Council of South Africa, The South Africa Sugar Association, Faculty of Community and Health Sciences; **Sweden:** Grants from the Swedish state under the Agreement concerning research and education of doctors; the Swedish Heart and Lung Foundation; the Swedish Research Council; the Swedish Council for Health, Working Life and Welfare, King Gustaf V:s and Queen Victoria Freemason’s Foundation, AFA Insurance; **Turkey:** Metabolic Syndrome Society, AstraZeneca, Sanofi Aventis; **United Arab Emirates:** Sheikh Hamdan Bin Rashid Al Maktoum Award For Medical Sciences and Dubai Health Authority, Dubai.

**Role of Sponsor:** The external funders and sponsors had no role in the design and conduct of the study; in the collection, analysis, and interpretation of the data; in the preparation, review, or approval of the

manuscript; or in the decision to submit the manuscript for publication.

**PURE Project Office Staff, National Coordinators, Investigators, and Key Staff:**

**Project office (Population Health Research Institute, Hamilton Health Sciences and McMaster University, Hamilton, Canada):** S Yusuf* (Principal Investigator).

S Rangarajan (Program Manager); K K Teo, S S Anand, C K Chow, M O’Donnell, A Mente, D Leong, A Smyth, P Joseph, M Duong, R D’Souza, M Walli-Attaei, S Islam (Statistician), W Hu (Statistician), C Ramasundarahettige (Statistician), P Sheridan (Statistician), S Bangdiwala, L Dyal, B Liu (Biometric Programmer), C Tang (Biometric Programmer), X Yang (Biometric Programmer), R Zhao (Biometric Programmer), L Farago (ICT), M Zarate (ICT), J Godreault (ICT), M Haskins (ICT), M Jethva (ICT), G Rigitano (ICT), A Vaghela (ICT), M Dehghan (Nutrition Epidemiologist), A Aliberti, A Reyes, A Zaki, B Connolly, B Zhang, D Agapay, D Krol, E McNeice, E Ramezani, F Shifaly, G McAlpine, I Kay, J Rimac, J Swallow, M Di Marino, M Jakymyshyn, M(a) Mushtaha, M(o) Mushtaha, M Trottier, N Aoucheva, N Kandy, P Mackie, R Buthool, R Patel, R Solano, S Gopal, S Ramacham, S Trottier

**Core Laboratories**: G Pare, M McQueen, S Lamers, J Keys (Hamilton), X Wang (Beijing, China), A Devanath (Bangalore, India).

**Argentina:** R Diaz*, A Orlandini, P Lamelas, M L Diaz, A Pascual, M Salvador, C Chacon; **Bangladesh:** O Rahman*, R Yusuf*, S A K S. Ahmed, T Choudhury, M Sintaha, A Khan, O Alam, N, Nayeem, S N Mitra, S Islam, F Pasha; **Brazil:** A Avezum*, C S Marcilio, A C Mattos, G B Oliveira; **Canada:**  K Teo***,** S Yusuf*****, Sumathy Rangarajan, A Arshad, B Bideri, I Kay, J Rimac, R Buthool, S Trottier, G Dagenais, P Poirier, G Turbide, AS Bourlaud, A LeBlanc De Bluts, M Cayer, I Tardif, M Pettigrew, S Lear, V de Jong, A N Saidy, V Kandola, E Corber, I Vukmirovich, D Gasevic, A Wielgosz, A Pipe, A Lefebvre, A Pepe, A Auclair, A Prémont, A S Bourlaud; **Chile:** F Lanas*, P Serón, M J Oliveros, F Cazor, Y Palacios; **China:** Liu Lisheng*, Li Wei*, Chen Chunming^#^, Zhao Wenhua. Hu Bo, Yin Lu, Zhu Jun, Liang Yan, Sun Yi, Wang Yang, Deng Qing, Jia Xuan, He Xinye, Zhang Hongye, Bo Jian, Wang Xingyu, Liu Xu, Gao Nan, Bai Xiulin, Yao Chenrui, Cheng Xiaoru, Wang Chuangshi, Li Sidong, Liu Weida, Lang Xinyue, Liu Xiaoyun, Zhu Yibing, Xie Liya, Liu Zhiguang, Ren Yingjuan, Dai Xi, Gao Liuning, Wang Liping, Su yuxuan, Han Guoliang, Song Rui, Cao Zhuangni, Sun Yaya, Li Xiangrong, Wang Jing, Wang Li, Peng Ya, Li Xiaoqing, Li Ling, Wang Jia, Zou Jianmei, Gao Fan, Tian Shaofang, Liu Lifu, Li Yongmei, Bi Yanhui, Li Xin, Zhang Anran, Wu Dandan, Cheng ying, Xiao Yize, Lu Fanghong, Li Yindong, Hou Yan, Zhang Liangqing, Guo Baoxia, Liao Xiaoyang, Chen Di, Zhang Peng, Li Ning, Ma Xiaolan, Lei Rensheng, Fu Minfan, Liu Yu, Xing Xiaojie, Yang Youzhu, Zhao Shenghu, Xiang Quanyong, Tang Jinhua, Liu Zhengrong, Qiang Deren, Li Xiaoxia, Xu Zhengting, Aideeraili.Ayoupu, Zhao Qian; **Colombia:** P Lopez-Jaramillo*, P A Camacho-Lopez, M Perez, J Otero-Wandurraga, D I Molina, C Cure-Cure, JL Accini, E Hernandez, E Arcos, C Narvaez, A Sotomayor, F Manzur, H Garcia, G Sanchez, F Cotes, A Rico, M Duran, C Torres; **India: Bangalore -** P Mony *, M Vaz*, S Swaminathan, AV Bharathi, K Shankar, A V Kurpad, K G Jayachitra, H A L Hospital, AR Raju, S Niramala, V Hemalatha, K Murali, C Balaji, A Janaki, K Amaranadh, P Vijayalakshmi, **Chennai** - V Mohan*, R M Anjana, M Deepa, K Parthiban, L Dhanasekaran, SK Sundaram, M Rajalakshmi, P Rajaneesh, K Munusamy, M Anitha, S Hemavathy, T Rahulashankiruthiyayan, D Anitha, R. Dhanasekar, S. Sureshkumar, D Anitha, K Sridevi, **Jaipur** - R Gupta, R B Panwar, I Mohan, P Rastogi, S Rastogi, R Bhargava, M Sharma, D Sharma, **Trivandrum** - V Raman Kutty, K Vijayakumar, S Nair, Kamala R, Manu MS, Arunlal AR, Veena A, Sandeep P Kumar, Leena Kumari, Tessi R, Jith S, K Ajayan, G Rajasree, AR Renjini, A Deepu, B Sandhya, S Asha, H S Soumya, **Chandigarh**- R Kumar, M Kaur, P V M Lakshmi, V Sagar J S Thakur, B Patro, R Mahajan, A Josh, G Singh, K Sharma, P Chaudary, **Iran:** R Kelishadi*, A Bahonar, N Mohammadifard, H Heidari, **Kazakhstan:** K Davletov*, B Assembekov, B Amirov; **Kyrgyzstan:** E Mirrakhimov*, S Abilova, U Zakirov, U Toktomamatov; **Malaysia: UiTM -** K Yusoff*, T S Ismail, K Ng, A Devi, N Mat-Nasir, AS Ramli, MNK Nor-Ashikin, R Dasiman, MY Mazapuspavina, F Ariffin, M Miskan, H Abdul-Hamid, S Abdul-Razak, N Baharudin, NMN Mohd-Nasir, SF Badlishah-Sham, MS Mohamed-Yassin, M Kaur, M Koshy, F A Majid, N A Bakar, N Zainon, R Salleh, SR Norlizan, NM Ghazali, M Baharom, H Zulkifli, R Razali, S Ali, CWJCW Hafar, F Basir; **UKM** - Noorhassim Ismail, M J Hasni, M T Azmi, M I Zaleha, R Ismail, K Y Hazdi, N Saian, A Jusoh, N Nasir, A Ayub, N Mohamed, A Jamaludin, Z Rahim; **Occupied Palestinian Territory:** R Khatib*, U Khammash, R Giacaman; **Pakistan:** R Iqbal*, R Khawaja, I Azam, K Kazmi; **Peru:** J Miranda*, A Bernabe Ortiz, W Checkley, R H Gilman, L Smeeth, R M Carrillo, M de los Angeles, C Tarazona Meza**;** **Philippines:** A Dans*, H U Co, J T Sanchez, L Pudol, C Zamora-Pudol, L A M Palileo-Villanueva, M R Aquino, C Abaquin, SL Pudol, K Manguiat, S Malayang; **Poland:** W Zatonski*, A Szuba, K Zatonska, R Ilow**^#^**, M Ferus, B Regulska-Ilow, D Różańska, M Wolyniec; **Saudi Arabia:** KF AlHabib*, M Alshamiri, HB Altaradi, O Alnobani, N Alkamel, M Ali, M Abdulrahman, R Nouri; **South Africa:** L Kruger^*^, A Kruger^#^, P Bestra, H Voster, A E Schutte, E Wentzel-Viljoen, FC Eloff, H de Ridder, H Moss, J Potgieter, A Roux, M Watson, G de Wet, A Olckers, J C Jerling, M Pieters, T Hoekstra, T Puoane, R Swart*, E Igumbor, L Tsolekile, K Ndayi, D Sanders, P Naidoo, N Steyn, N Peer, B Mayosi^#^, B Rayner, V Lambert, N Levitt, T Kolbe-Alexander, L Ntyintyane, G Hughes, J Fourie, M Muzigaba, S Xapa, N Gobile , K Ndayi, B Jwili, K Ndibaza, B Egbujie; **Sweden** A Rosengren*, K Bengtsson Boström, A Rawshani, A Gustavsson, M Andreasson, L Wirdemann; **Tanzania:** K Yeates*, M Oresto, N West **Turkey:** A Oguz*, N Imeryuz, Y Altuntas, S Gulec, A Temizhan, K Karsidag, K B T Calik, A K Akalin, O T Caklili, M V Keskinler, K Yildiz; **United Arab Emirates:** A H Yusufali, F Hussain, M H S Abdelmotagali, D F Youssef, O Z S Ahmad, F H M Hashem, T M Mamdouh, F M AbdRabbou, S H Ahmed, M A AlOmairi, H M Swidan, M Omran, N A Monsef ; **Zimbabwe:** J Chifamba*, T Ncube, B Ncube, C Chimhete, G K Neya, T Manenji, L Gwaunza, V Mapara, G Terera, C Mahachi, P Murambiwa, R Mapanga, A Chinhara

*National Coordinator

^#^ Deceased

**PURE Country Institution Names:**

|  | **Institution** |
| --- | --- |
| **South Africa** | Faculty of Health Science  North-West University  Potchefstroom Campus |
|  | University of the Western Cape  Department of Dietetics and Nutrition  Private Bag X17, 7535  Bellville, South Africa |
| **Zimbabwe** | University of Zimbabwe  College of Health Sciences  Physiology Department  Harare, Zimbabwe |
| **Tanzania** | Pamoja Tunaweza Health Research Centre, Moshi, Tanzania  Division of Nephrology, Department of Medicine  Queen's University |
| **China** | National Centre for Cardiovascular Diseases  Cardiovascular Institute & Fuwai Hospital  Chinese Academy of Medical Sciences  167, Bei Li Shi Lu, Beijing, China |
|  | Fuwai Hospital  167 Beilishi Rd. Xicheng District  Beijing. 100037 China |
| **Philippines** | University of Philippines, Section of Adult Medicine & Medical Research Unit, Manila, Philippines |
| **Pakistan** | Department of Community Health Sciences and Medicine  Aga Khan University  Stadium Road, P.O Box 3500  Karachi Pakistan |
| **India, Bangalore** | St John's Medical College and Research Institute Bangalore 560034, India |
| **India, Chennai** | Madras Diabetes Research Foundation &  Dr. Mohan’s Diabetes Specialities Centre, Chennai |
| **India Jaipur** | Eternal Heart Care Centre and Research Institute, Jaipur |
| **India, Trivandrum** | Health Action by People,  Thiruvananthapuram, Kerala, 695011 INDIA |
| **India, Chandigarh** | School of Public Health, Post Graduate Institute of Medical Education & Research, Chandigarh (India) |
| **Bangladesh** | Independent University, Bangladesh  Bashundhara, Dhaka  Bangladesh |
| **Malaysia** | Universiti Teknologi MARA, Sungai Buloh, Selangor, Malaysia AND UCSI University, Cheras, Selangor, Malaysia |
|  | Department of Community Health. Faculty of Medicine. University Kebangsaan Malaysia. Kuala Lumpur. Malaysia |
| **Poland** | Wroclaw Medical University Department of Internal Medicine; Department of Social Medicine Borowska 213 street; 50- 556 Wroclaw, Poland |
|  | Department of Epidemiology,  The Maria Skłodowska-Curie Memorial Cancer Center and Institute of Oncology  02-034 Warsaw, 15B Wawelska str.  Poland |
| **Turkey** | Istanbul Medeniyet University  Istanbul, Turkey |
| **Sweden** | Sahlgrenska Academy  University of Gothenburg  Sweden |
| **Iran** | Isfahan Cardiovascular Research Center, Isfahan Research Institute  Isfahan University of Medical Sciences, Isfahan, Iran |
| **UAE** | Dubai Medical University, Hatta Hospital, Dubai Health Authority, Dubai, United Arab Emirates |
| **Saudi Arabia** | Department of Cardiac Sciences, King Fahad Cardiac Center  College of Medicine  King Saud University  Riyadh, Saudi Arabia |
| **Palestine** | Institute of Community and Public Health, Birzeit University, Ramallah, occupied Palestinian territory |
| **Canada** | Université Laval Institut universitaire de cardiologie et de pneumologie de Québec, Quebec  Canada G1V 4G5 |
|  | Simon Fraser University,  Dept. of Biomedical Physiology & Kinesiology, BC, Canada |
|  | Department of Medicine,  University of Ottawa,  Ottawa, Canada |
|  | Population Health Research Institute, McMaster University, Hamilton Health Sciences, Hamilton, Ontario, Canada |
| **Argentina** | Estudios Clinicos Latinoamerica ECLA  Rosario, Santa Fe  Argentina  Department of Chronic Diseases  South American Center of Excellence for Cardiovascular Health (CESCAS)  Institute for Clinical Effectiveness and Health Policy (IECS) |
| **Brazil** | International Research Center, Hospital Alemão Oswaldo Cruz  São Paulo, SP  Brazil |
| **Colombia** | Facultad de Ciencias de la Salud, Universidad de Santander (UDES), Bucaramanga, Santander,  Fundacion Oftalmologica de Santander (FOSCAL)  Floridablanca-Santander, Colombia |
| **Chile** | Universidad de La Frontera  Temuco, Chile |
| **Ecuador** | DECANO  Facultad de Ciencias de la Salud Eugenio Espejo  Universidad Tecnológica Equinoccial  Dirección: Av. Mariscal Sucre s/n y Av. Mariana de Jesús, Quito Ecuador |
| **Peru** | CRONICAS Centro de Excelencia en Enfermedades Crónicas | [www.cronicas-upch.pe](http://www.cronicas-upch.pe/)  Universidad Peruana Cayetano Heredia | www.upch.edu.pe  Av. Armendáriz 497, Miraflores, Lima |
| **Russia** | Research Institute for Complex Issues of Cardiovascular Diseases, Kemerovo, Russia  Institute For Medical Education, Yaroslav-the-Wise Novgorod State University Ministry of Education and Science of the Russian Federation  Russia, Saint-Petersburg, 197022,  Karpovka river emb., Bld.13, office 28 |
| **Kazakhstan** | Research Institute of Cardiology & Internal Diseases, Almaty, Kazakhstan |
| **Kyrgyzstan** | Kyrgyz Society of Cardiology, National Center of Cardiology and Internal Disease, Bishkek, Kyrgyzstan |

Appendix-

Supplementary Figure 1- Scatterplot of GNI Per Capita versus Proportion Whose Finances Were Adversely Impacted, by Country


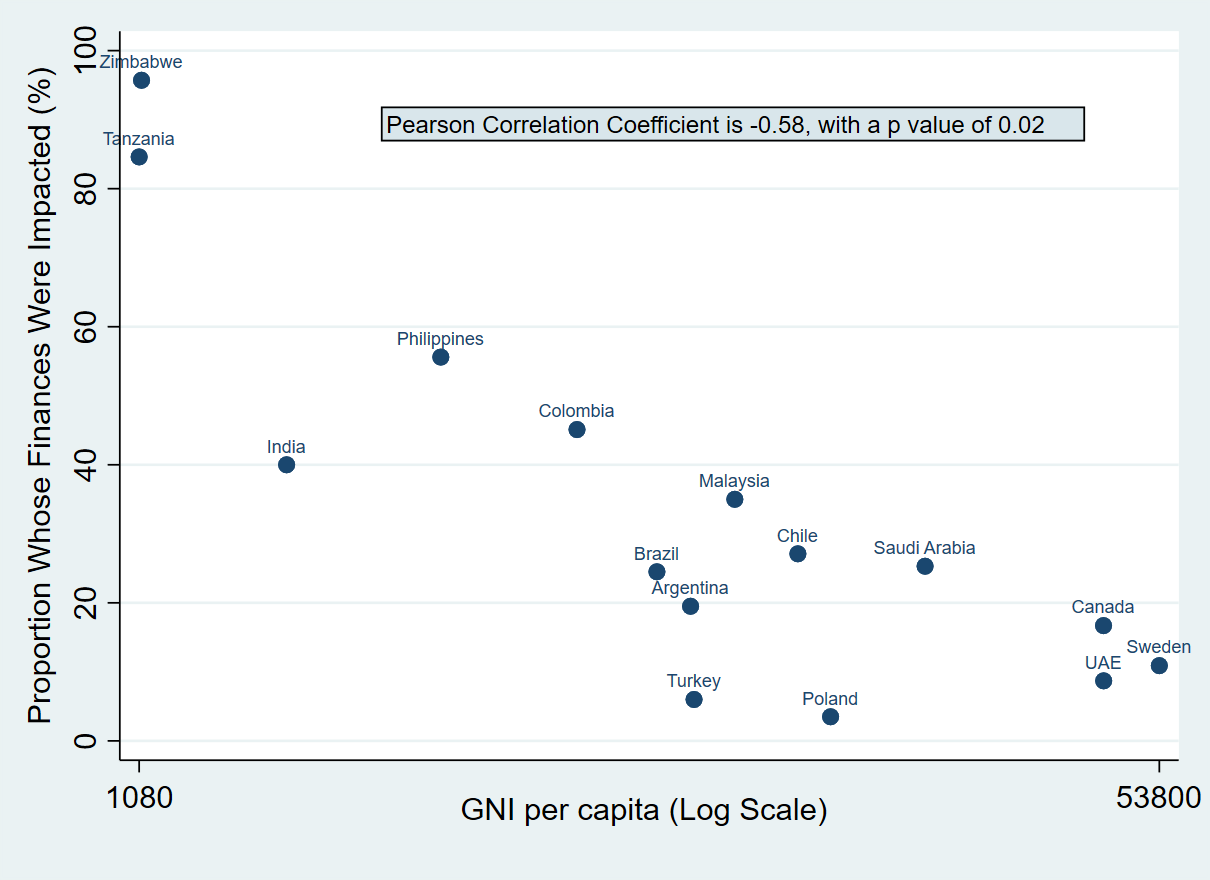


[GNI per capita is from 2020 World Bank Data and uses the Atlas method (shown in current US$).](https://data.worldbank.org/indicator/NY.GNP.PCAP.CD)  South Africa excluded from this figure as it had <100 participants.

Supplementary Figure 2- Scatterplot of GNI Per Capita versus Vaccination Rates, by Country


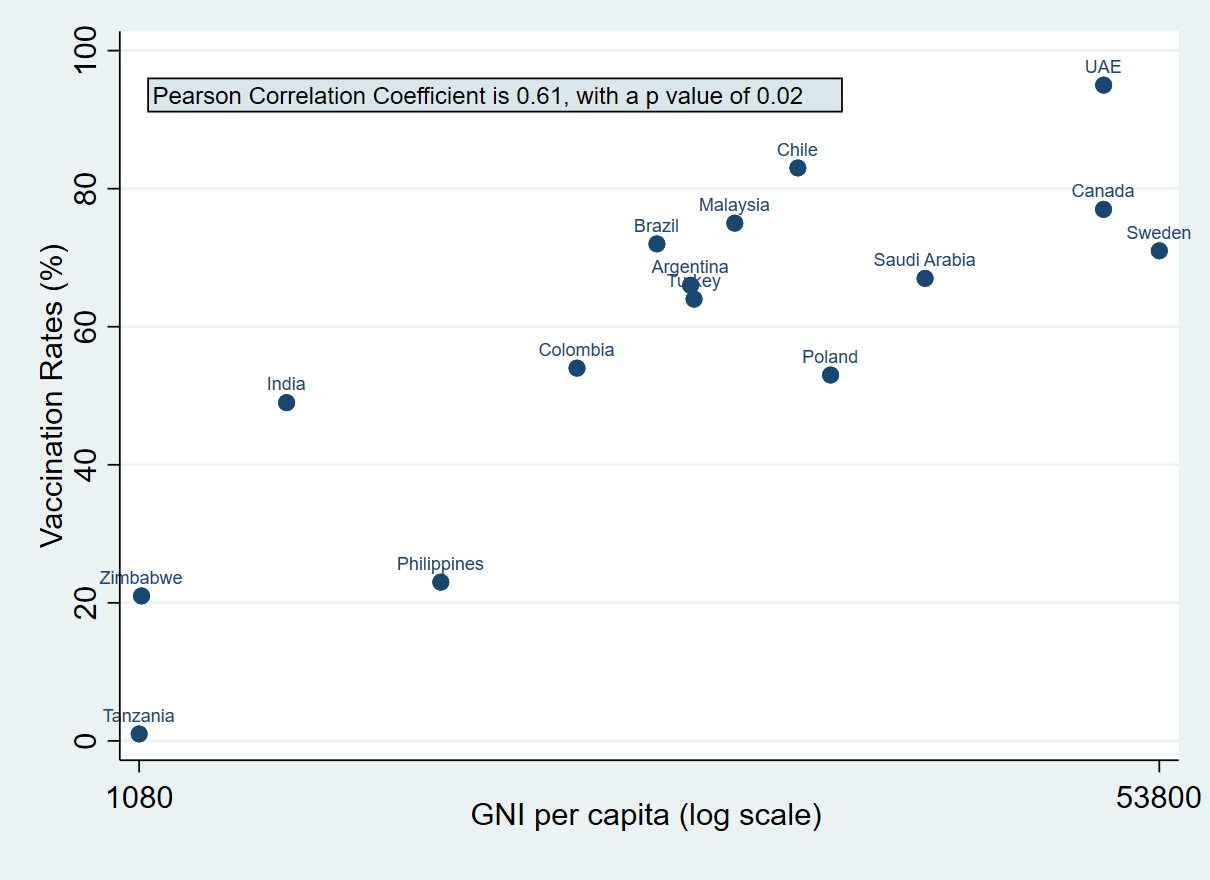


South Africa excluded from this figure as it had <100 participants. [GNI per capita is from 2020 World Bank Data and uses the Atlas method (shown in current US$). Vaccination Rates represent the proportion of a country’s total population that received at least 1 dose of a COVID-19 vaccine (as of 11](https://data.worldbank.org/indicator/NY.GNP.PCAP.CD)^th^ October, 2021).

Supplementary Figure 3- Scatterplot of Vaccination Rates versus Proportion Whose Finances Were Adversely Impacted, by Country


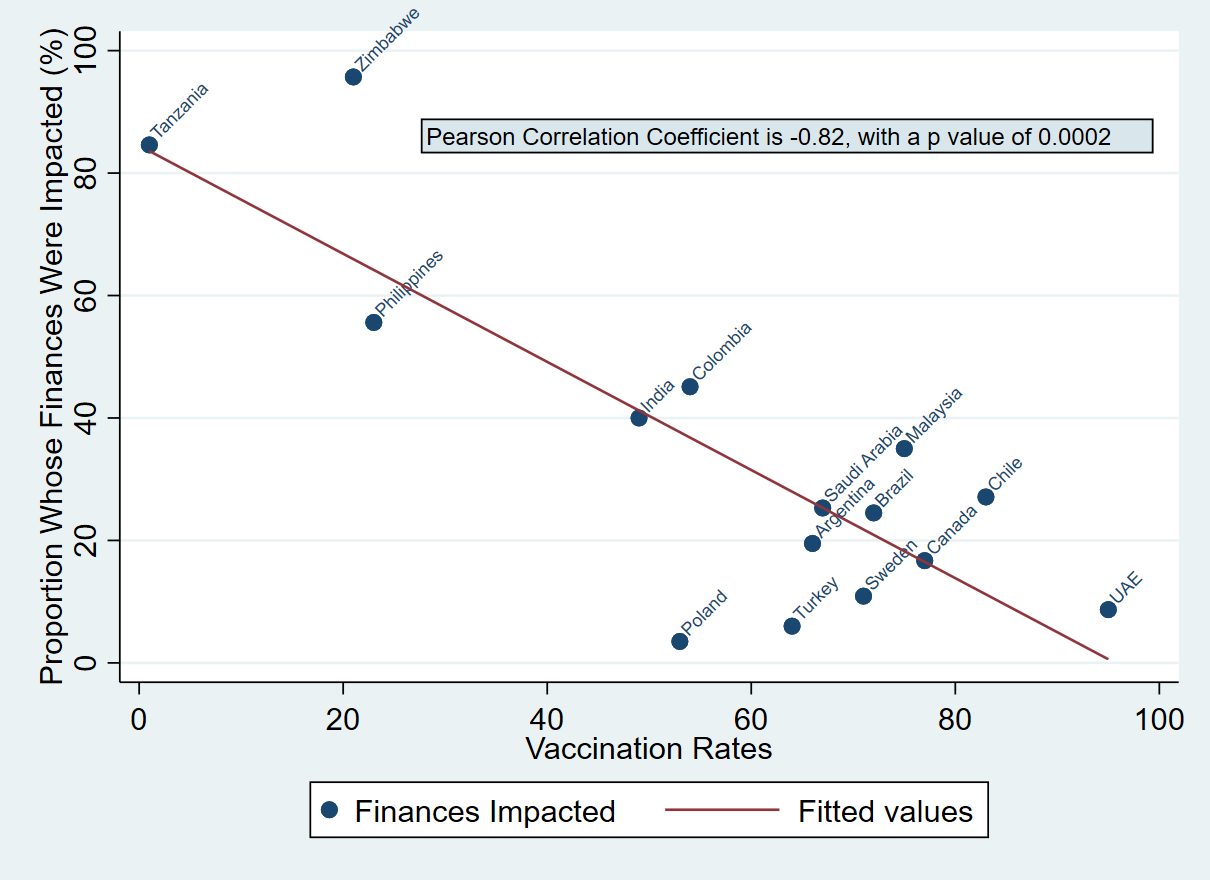


Vaccination Rates represent the [proportion of a country’s total population that received at least 1 dose of a COVID-19 vaccine (as of 11](https://data.worldbank.org/indicator/NY.GNP.PCAP.CD)^th^ October, 2021). South Africa excluded from this figure as it had <100 participants.
